# Asymmetric chromosome segregation in *Xanthomonas citri* ssp. *citri*

**DOI:** 10.1002/mbo3.145

**Published:** 2013-12-15

**Authors:** Amanda P Ucci, Paula M M Martins, Ivy F Lau, Maurício Bacci, José Belasque, Henrique Ferreira

**Affiliations:** 1 Depto. de Ciências Biológicas, Faculdade de Ciências Farmacêuticas, Universidade Estadual PaulistaRodovia Araraquara/Jaú Km 1, CP 502, Araraquara, São Paulo, 14801-902, Brazil; 2 Depto. de Bioquímica e Microbiologia, Instituto de Biociências, Universidade Estadual PaulistaAv. 24A, 1515, Rio Claro, São Paulo, 13506-900, Brazil; 3 Fundo de Defesa da CitriculturaAv. Dr. Adhemar P. de Barros, 201, Araraquara, São Paulo, 14807-040, Brazil

**Keywords:** Cell division arrest, chromosome segregation, citrus canker.

## Abstract

This study was intended to characterize the chromosome segregation process of *Xanthomonas citri* ssp. *citri* (Xac) by investigating the functionality of the ParB factor encoded on its chromosome, and its requirement for cell viability and virulence. Using TAP tagging we show that ParB is expressed in Xac. Disruption of *parB* increased the cell doubling time and precluded the ability of Xac to colonize the host citrus. Moreover, Xac mutant cells expressing only truncated forms of ParB exhibited the classical phenotype of aberrant chromosome organization, and seemed affected in cell division judged by their reduced growth rate and the propensity to form filaments. The ParB-GFP localization pattern in Xac was suggestive of an asymmetric mode of replicon partitioning, which together with the filamentation phenotype support the idea that Xac may control septum placement using mechanisms probably analogous to *Caulobacter crescentus*, and perhaps *Vibrio cholerae*, and *Corynebacterium glutamicum*. Xac exhibits asymmetric chromosome segregation, and the perturbation of this process leads to an inability to colonize the host plant.

## Introduction

Citrus canker is a serious disease present in the major citrus-producing areas around the world. Currently, there are no effective curative measures to safeguard the orchards, where the eradication of affected trees is the only reliable method to prevent the spread of the causal agent Xac to regions where it has not been detected (Gottwald et al. 2002). The eradication of plants is a highly controversial, costly, and difficult method to control citrus canker. In addition, its effectiveness is only possible in areas with very low incidence of the disease (Belasque et al. 2010). Other control measures have been adopted in areas where citrus canker is endemic or the eradication of affected trees is not mandatory. These methods, collectively known as the disease management approach, include distinct cultivation strategies, the use of chemical formulations to contain the spread of Xac, and the plantation of citrus genotypes displaying relative resistance to citrus canker. The disease management approach is therefore intended to minimize and/or prevent damages and losses promoted by citrus canker, such as blemish on fruits, symptomatic fruit drop, defoliation, and stem dieback (Behlau et al. 2010).

Citrus canker has an endemic status in some of the main citrus-producing areas in the world, the state of Florida, USA, and in the southern portion of South America. In Florida, the eradication program was abandoned in January 2006, and as a consequence, citrus canker is spreading throughout the region. In both areas, growers adopt the disease management system. On the other hand, the state of São Paulo, Brazil, still keeps an eradication program with mandatory elimination of foci of the disease; however, due to a diminished effort to detect and eradicate infected trees, citrus canker is becoming an epidemic (see references within Ferreira and Belasque 2011). As a result, in some years growers may have to manage citrus canker in the state of São Paulo as it is already done in the southern states of Brazil. For these reasons in depth knowledge of the biology of Xac may help in the development of strategies to control and minimize the losses caused by this plant pathogen.

Recently, we characterized some esters of gallic acid that interfere with the subcellular localization of ParBgreen fluorescent protein (GFP) and also GFP-ZapA in Xac (Silva et al. 2013). As ParB and ZapA are components of the bacterial chromosome segregation (segrosome) and cell division (divisome) machineries (Gueiros-Filho and Losick 2002; Mierzejewska and Jagura-Burdzy 2012), respectively, alkyl gallates are believed to target components of the segrosome and/or the divisome (Silva et al. 2013). ParB-like factors are DNA-binding proteins encoded on plasmids or on the chromosomes of many bacteria (Leonard et al. 2005b; Gerdes et al. 2010; Mierzejewska and Jagura-Burdzy 2012). They act together with cognate ATPases, ParA-like factors, to operate efficient replicon segregation in prokaryotes. Segregation of low copy number plasmids and chromosomes requires the specific interaction of ParB with short *cis-*acting elements designated *parS*, which on bacterial chromosomes are normally located around the origins of replication (Livny et al. 2007). Homology searches showed that Xac possesses at least one of these *cis* elements (TGTTCCACGTGGAACG; genomic coordinates c2753..2768), which is located right at the 3′-end of the *dnaN* gene on the bacterial chromosome (Livny et al. 2007). Active segregation of ParB–*parS* complexes (also known as the bacterial centromeres), and consequently the origins of replication, occurs with the help of ParA filaments that help to orient newly replicated chromosomes to opposite positions within the cells, a mechanism elegantly demonstrated for the models *Vibrio cholerae* (*V. cholerae*) and *Caulobacter crescentus* (*C. crescentus*) (Fogel and Waldor 2006; Ptacin et al. 2010). In the case of ZapA, this protein is well conserved among *Bacteria*, and shows septal localization dependent on FtsZ (Gueiros-Filho and Losick 2002). ZapA stimulates FtsZ polymer bundling, as well as the cross-linking of FtsZ filaments, which is believed to stabilize the Z-ring during cytokinesis (Gueiros-Filho and Losick 2002; Low et al. 2004; Mohammadi et al. 2009; Dajkovic et al. 2010).

The fact that alkyl gallates are able to target at once chromosome segregation and cell division in Xac is supported by demonstrations that these processes are interlinked in other bacteria, for example, *C. crescentus*, *Streptomyces coelicolor* (*S. coelicolor*), *Mycobacterium smegmatis* (*M. smegmatis*), and *Corynebacterium glutamicum* (*C. glutamicum*) (Easter and Gober 2002; Thanbichler and Shapiro 2006a; Jakimowicz et al. 2007; Donovan et al. 2010; Ginda et al. 2013). In *S. coelicolor,* for instance, ParAB is required for proper distribution of chromosomal copies within the aerial hyphae during sporulation (Jakimowicz et al. 2005, 2007). ParB bound to the *oriC* region organizes segregation complexes, while ParA coordinates their spatial distribution for a concomitant assembly of multiple septa. Therefore, lack of ParA interferes not only with chromosome segregation but also with septum placement in *S. coelicolor* (Jakimowicz et al. 2007). In *C. crescentus*, MipZ, an inhibitor of FtsZ polymerization, follows ParB/*parS* precluding the formation of the Z-ring at sites other than in the middle of the cells (Thanbichler and Shapiro 2006a; Kiekebusch et al. 2012). In a recent model, ParB/DNA was proposed to catalyze the conversion of MipZ monomers into the active/dimeric inhibitor of FtsZ, and given the polar localization of ParB/DNA in *C. crescentus*, a cloud of active inhibitor is formed and surrounds the polar regions of the cells (Kiekebusch et al. 2012). In the case of *M. smegmatis*, deletion of *parA* produced aberrant septum placement (Ginda et al. 2013); this phenotype was attributed to overall chromosome disorganization, and it was suggested that another factor, maybe analogous to MipZ, could link both processes. Finally, *C. glutamicum* possesses an orphan *parA*-like gene that codes for a protein designated PldP (ParA-like division protein) (Donovan et al. 2010). Mutation of *pldP* had just a mild effect on segregation producing a slight increase in the number of anucleate cells, and overexpression led to cell elongation; however, a fluorescent form of PldP localized close to the septum, and protein interaction analysis showed that PldP associated with both ParB and ParA, making it a possible candidate for a septum inhibitor.

Here, we report on the role of ParB in the chromosome segregation process of Xac. Using a tandem affinity purification (TAP)-TAG version of ParB (ParB-TAP), we demonstrate that this protein is stably expressed in Xac. Disruption of ParB from Xac severely compromised cell division and the ability to induce disease symptoms *in planta*. Subsequent analyses of the ParB-GFP subcellular localization pattern coupled to the observation that chromosome segregation and cell division may be interlinked in this plant pathogen raised the hypothesis that the strategies used by Xac to deal with these tasks resembles those that have been described for the model organism *C. crescentus* (Thanbichler and Shapiro 2006b).

## Materials and Methods

### Bacterial strains and plasmids

The strains and plasmids used are listed in Table 1; oligonucleotides are listed in Table S1. *E. coli* was cultivated at 37°C in luria broth (Sambrook et al. 1989); Xac was cultivated at 30°C in nutrient yeast glycerol (NYG) (Daniels et al. 1984). The TAP-tag expression vector pHF5Ca (**FJ562210**) is a variant of pPM2a (Martins et al. 2010). To construct pHF5Ca, we substituted the *gfpmut1* cassette of pPM2a by the *tap1479*, which was extracted from pBS1479 (Puig et al. 2001) using PCR with primers PBS1479F/PBS1479R; *gfpmut1* was released from pPM2a by a *Bam*HI/*Xba*I digest, whereas *tap1479* was treated with the same enzymes prior to ligation. The ParB-TAP expression vector, pAPU1, was constructed by the ligation of *parB* (PCR amplified from the chromosome of Xac with primers ParBF20070822/ParBR20080530) into the *Bam*HI site of pHF5Ca. pAPU2 was generated by the ligation of *parB124-769* (PCR amplified using primers parBintF/parBintR), into the cloning vector pCR-2.1-TOPO (Invitrogen, Grand Island, NY). The ParB-GFP expression plasmid, pAPU3, is a derivative of pPM7g, which carries the *parB* gene (PCR amplified with primers ParBF20070822/ParB20100309) ligated between *Bam*HI/*Xho*I restriction sites.

**Table 1:** Strains and plasmids.

	Relevant characteristics^1^	References
*Strain*		
* Xanthomonas citri* ssp. *citri*	IBSBF 1594; formerly known as *Xanthomonas axonopodis* pv. citri strain 306 (Xac); Ap^R^	da Silva et al. (2002), Schaad et al. (2005, 2006)
* E. coli* DH10B	Cloning strain	Invitrogen
* E. coli* BL21(DE3)	Protein expression strain (pET system)	Novagen
Xac *amy*::pAPU1	pHF5Ca-*parB* integrated into the *amy* locus of Xac; Ap^R^ Km^R^	This work
Xac *parB*::pAPU1	pHF5Ca-*parB* integrated in *parB*; Ap^R^ Km^R^	
Xac *parB*::pAPU2	pCR-2.1-TOPO-*parB124-769* integrated in *parB*; Ap^R^ Km^R^	
Xac *parB*::pAPU3	pPM7g-*parB* integrated in *parB*; Ap^R^ Km^R^	
*Plasmids*		
pPM2a and pPM7g	GFP expression vectors; *xylR pxyl gfpmut1 bla neo*	Martins et al. (2010)
pHF5Ca	TAP-tag expression vector; *xylR pxyl tap1479 bla neo*	This work
pAPU1	pHF5Ca-*parB*: *xylR pxyl parB-tap1479 bla neo*	
pAPU2	Derivative of pCR-2.1-TOPO (Invitrogen) carrying *parB124-769*; *bla neo*	
pAPU3	pPM7g-*parB*: *xylR pxyl parB-gfpmut1 bla neo*	

1 Ap^R^ and Km^R^, ampicillin and kanamycin resistance, respectively; *bla* and *neo*, genes for *β*-lactamase and Neomycin phosphotransferase, respectively.

### General methods

Electrotransformation of Xac was followed (Ferreira et al. 1995). Southern and Western blot analyses were carried out following the instructions contained into the DIG (Roche, Indianapolis, IN) and the Amersham (GE, Fairfield, CT) ECL Western blotting kits, respectively. To detect ParB-TAP we used a horseradish peroxidase-conjugated anti-horse (IgG) raised in rabbits as an unique antibody (Sigma-A6917, Sigma-Aldrich, St. Louis, MO).

### Pathogenicity tests

The host plant used was Rangpur lime (*C. limonia* Osbeck). Citrus plants were cultivated under greenhouse conditions at 25–35°C. For the infiltration tests, Xac cells were cultivated in NYG medium until the OD_600 nm_ of ˜1. Cells were subsequently diluted to 10^5^ colony forming unit (CFU)/mL in PBS 1X, and infiltrated on the abaxial surface of leaves using hypodermic syringes without needles. Symptoms were observed during the course of 60 days. All the tests were performed in triplicates.

### Microscopy

Wild-type and mutant strains of Xac were immobilized onto agarose-covered slides for microscope observations as previously described (Martins et al. 2010). Cells were visualized using an Olympus BX-61 microscope and documented with a monochromatic XM-10 camera. Image processing and analyses were conducted using the software Cell^F (Olympus, Center Valley, PA).

### Statistics

Statistical analysis was performed using one-way analysis of variance (ANOVA) followed by Tukey posttest (*P* < 0.05).

## Results

### ParB is expressed in Xac

**Figure 1:**
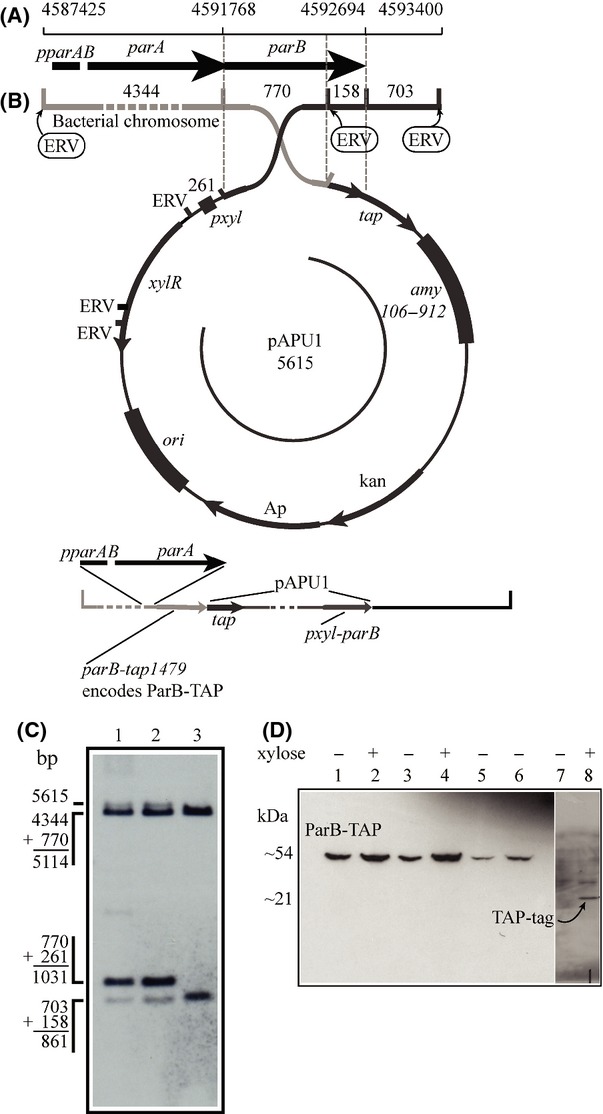
Activity of the Xac *parAB* operon. The activity of the *parAB* operon was demonstrated by the ability of Xac *parB*::pPAU1 mutants to express ParB-TAP under the control of the native *parAB* promoter. The ParB-TAP expression vector was integrated into the *parB* locus of Xac by a single crossover event (B) generating the genomic structure depicted at the bottom of (B). (A) Genomic coordinates of the *parAB* operon. (B) Schematics of the integration of pPAU1 (carrying the *parB-tap* fusion) into the *parB* locus. Numbers above the map and around the circle indicate the sizes in base-pairs of the DNA fragments delimited; ERV, *Eco*RV restriction sites. (C) Southern blot analysis of Xac *parB*::pPAU1 mutants: genomic DNA was digested with *Eco*RV and subsequently probed with a DIG-labeled *parB*. The sizes of the DNA fragments detected correspond to those illustrated in (B). Lanes 1 and 2, Xac *parB*::pPAU1 mutants 1 and 2, respectively; lane 3, wild-type Xac. (D) Western blot was used to detect ParB-TAP (54 kDa) in the protein extracts of Xac *parB*::pPAU1 and Xac *amy*::pPAU1 mutants. Lanes: 1–2, Xac *amy*::pPAU1 mutant 1; 3–4, Xac *amy*::pPAU1 mutant 2; 5, Xac *parB*::pPAU1 mutant 1; 6, Xac *parB*::pPAU1 mutant 2; 7, Xac wild type (negative control); 8, Xac *amy*::pHF5Ca (expresses only the TAP tag of 21 kDa). The inductor xylose was added as indicated.

The annotation of the genome sequence of Xac showed the presence of at least five *parA*-like open reading frames (ORFs) with the following designations: XAC0192, XAC1907, XAC2205, XAC2433, and XAC3905. The last one, XAC3905, was found in a small operon (*parAB*) with XAC3906, which was identified as a chromosomal *parB* homologue (da Silva et al. 2002). In order to verify if *parB* was expressed in Xac, we monitored the activity of the *parAB* promoter (*pparAB*) by the ability of a Xac mutant strain to express a TAP-tagged version of ParB (ParB-TAP). This mutant was prepared by the integration of the suicide plasmid pAPU1 (carrying the fusion *parB-tap1479*) by a single crossover event into the *parB* locus of Xac (Xac *parB*::pPAU1) using an established protocol (Martins et al. 2010). Following pAPU1 integration, *parB* was duplicated, where the native copy of the gene was adjacent to the xylose promoter (*pxyl*) carried by the plasmid, whereas the *parB-tap1479* fusion had its expression governed by the native promoter of the *parAB* operon (Fig. 1B). The genomic structure of two selected Xac *parB*::pPAU1 mutants was evaluated by Southern blot using the *parB* gene as a probe (1C), and the detection of the hybridization bands of 1.031 bp and 5.615 bp confirmed the integration of pAPU1 into the *parB* locus (compare lanes 1–3 and 2–3). To detect the production of ParB-TAP by Xac *parB*::pPAU1, cells were cultivated in NYG medium until the mid-log phase, and subsequently processed for the immune detection of the TAP-tagged protein by Western blot (Fig. 1D). A signal of ˜54 kDa was observed for the two mutant strains analyzed, which is consistent with the size expected for ParB-TAP (lanes 5–6). No bands could be detected for the wild-type strain (lane 7), whereas Xac transformed with the empty vector (Xac *amy*::pHF5Ca) produced a signal relative to the TAP tag of ˜21 kDa (lane 8). We also included in these analyses two mutants in which pAPU1 had integrated into the *amy* locus through recombination between the *amy106-912* fragment carried by pAPU1 and the *α*-amylase gene of Xac (Xac *amy*::pPAU1), hence, they express ParB-TAP ectopically to serve as a control. Note that both Xac *amy*::pPAU1 mutants also express ParB-TAP, but at higher levels (compare lanes 1 and 3 with 2 and 4 for noninduced and xylose-induced cultures, respectively, with lanes 5–6). The lower expression of ParB-TAP by the Xac *parB*::pPAU1 mutants is probably due to the fact that these cells have *parB-tap1479* under the control of *pparAB*, and therefore, they express ParB-TAP at physiological/natural levels. In summary, the stable production of ParB-TAP from the native *parAB* promoter confirmed the activity of the *parAB* operon in Xac.

### Xac parB mutant is unable to induce citrus canker symptoms

**Figure 2:**
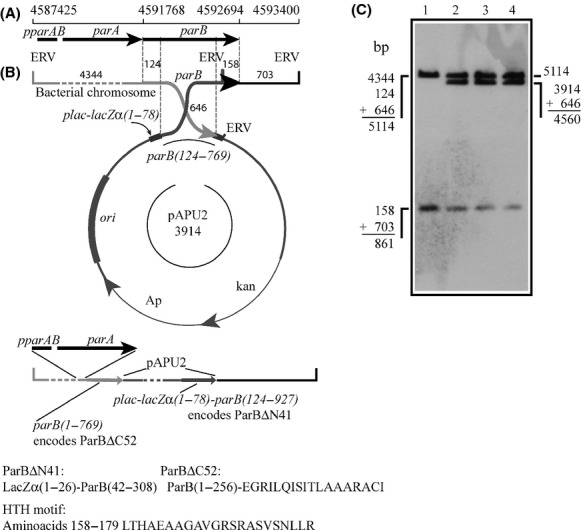
The strategy used to disrupt *parB* in Xac. The suicide vector pPAU2, which carries the *parB124–769* fragment, was integrated into the *parB* locus of Xac by a single crossover event; integration generated the genomic structure depicted underneath the vector in which ParBΔ52 can be expressed under the control of the native promoter of ParB (*pparAB*). (A) Genomic coordinates of *parAB*. (B) Schematics of integration: upon plasmid insertion, two new forms of *parB* will be generated: one coding for ParBΔN41, a fusion between the first 26 amino acids of LacZα (coded by the expression vector) plus residues 42–308 of ParB, and another that codes for ParBΔC52, which lacks the 52 C-terminal residues of ParB and has an addition of the 18 new amino acids shown. Both truncated ParB proteins still carry the helix-turn-helix (HTH) motif shown. The numbers above the map and around the circle indicate the sizes of the DNA fragments delimited; the predicted helix-turn-helix motif of ParB is depicted below the drawings; ERV, *Eco*RV restriction sites. (C) Southern blot analysis: total DNA of the three Xac *parB*::pAPU2 mutants was digested with *Eco*RV, and probed with *parB*. The sizes of the hybridization fragments detected correspond to those estimated based on the genomic coordinates shown in B.

Several unsuccessful attempts were made to delete the *parB* gene of Xac. To circumvent this problem and to be able to extend our functional characterization of the *parAB* operon of Xac, we decided to construct a mutant strain that can only express truncated forms of ParB. This was achieved by transforming Xac with the suicide vector pAPU2 that carried a DNA fragment corresponding to *parB124-769*. Upon integration into the *parB* locus (Fig. 2B) two new forms of *parB* should arise: one under the control of *pparAB*, which lacks the coding sequence for the 52 C-terminal residues of ParB (codes for an equivalent of ParBΔC52, and that has a DNA addition from the vector coding for extra 18 residues not related to LacZ [Fig. 2B]), and another that can only be expressed by the activity of the *plac* promoter of pPAU2, and that codes for a LacZ*α*-ParB chimera (LacZ*α*1-26-ParB42-308, which corresponds to a ParB deleted for the first 41 N-terminal aminoacids, ParBΔN41). Although ParBΔC52 carries a predicted HTH motif (Fig. 2B; residues 158–179 identified using GYM2.0; Narasimhan et al. 2002), this protein is not expected to form dimers and should be impaired in DNA binding (by analogy to data from the biochemical characterization of Spo0JΔ20, and also of a C-terminal deletion form of ParB from *Thermus thermophilus* (Leonard et al. 2004; Murray et al. 2006); and H. Ferreira and J. Errington, unpublished data). On the other hand, ParBΔN41, if correctly expressed and folded, would be compromised in its ability to interact and/or stimulate the ParA partner as demonstrated for the ParAB system of *T. thermophilus* (Leonard et al. 2005a).

Upon transformation of Xac with pAPU2, and the expected integration of this plasmid into the bacterium chromosome, the genomic context surrounding the *parB* locus of three independently selected Xac *parB*::pAPU2 mutants, which were derived from the same disruption event, was checked by Southern blot (Fig. 2C), where the observation of the diagnostic hybridization band of 4.560 bp confirmed the alterations. The three mutant strains exhibited a slower growth rate when compared with the wild-type strain. Growth curves of these mutants displayed a slower doubling time on the first 24 h, being only able to reach the usual culture limit for Xac (OD_600 nm_ ˜2) after 30 h of growth. This contrasts with the 18 h normally taken by the wild-type strain to approach the growth peak (Figure S1). Note that it took twice as much for the mutant to get to the same culture limit of OD_600 nm_ ˜2. Furthermore, the recovery of kanamycin-resistant mutants after transformation took almost 5 days (120 h), whereas wild-type colonies become visible in up to 48 h.

**Figure 3:**
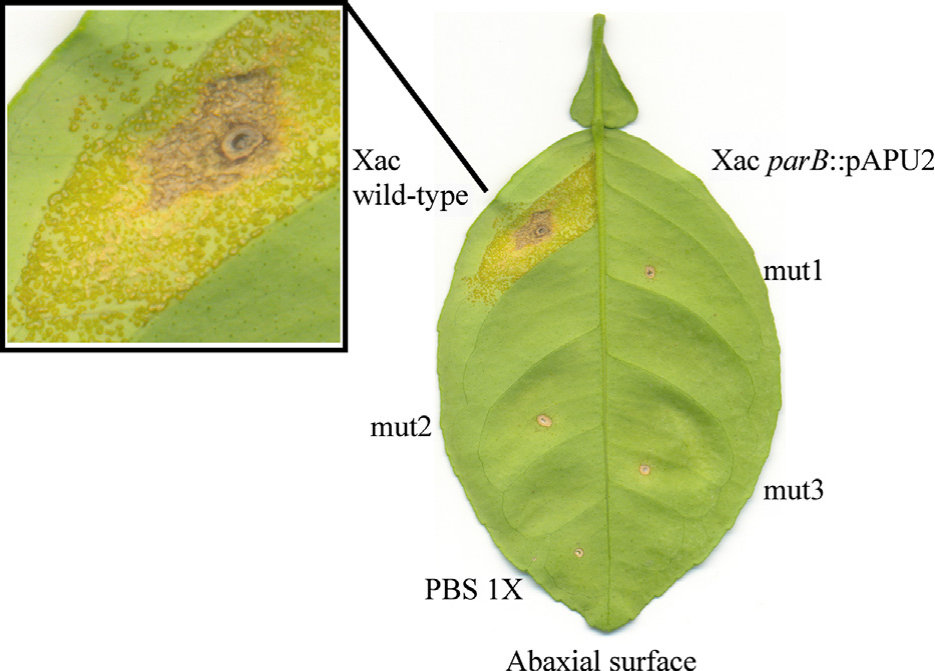
Xac mutants disrupted for *parB* are unable to colonize the host citrus. Three Xac *parB*::pPAU2 mutants were inoculated in leaves of Rangpur lime alongside the wild-type Xac and the diluent PBS 1X. Only Xac wild type was capable of inducing the typical symptoms of citrus canker as eruptive lesions within chlorotic spaces. Infected plants were kept in green house for a period of 60 days in order to score for the appearance of citrus canker symptoms. Here, we show a representative experiment; tests were performed in triplicates.

As a plant pathogen, the ability to colonize its host is a vital process for Xac. Since the disruption of *parB* altered the growth pattern of the Xac *parB*::pAPU2 mutants on rich medium, we tested whether these cells would still be able to colonize citrus plants. The three Xac *parB*::pAPU2 mutants were infiltrated in leaves of Rangpur lime alongside the wild-type strain. While the region inoculated with the wild-type strain evolved erumpent–corky and brownish lesions surrounding the infiltration point, none of the mutant strains was capable of inducing the typical symptoms of citrus canker (Fig. 3), even after the long incubation periods of 60 days. We conclude that disruption of *parB* in Xac retarded the cell doubling time and promoted a loss of the ability to colonize the host citrus.

### Alteration of the normal ParB function disrupts cell division in Xac

Disruption, deletion, and/or the overexpression of ParB-like proteins in several microorganisms, including *C. crescentus*, *C. glutamicum*, and *Pseudomonas aeruginosa* (*P. aeruginosa*), may lead to pleiotropic effects such as cell filamentation, loss of motility, and perturbations in the basic processes of chromosome segregation and cell division (Mohl and Gober 1997; Bartosik et al. 2009; Donovan et al. 2010). In this work, the inability to colonize citrus observed for Xac *parB*::pAPU2 could be a consequence of at least two other deficiencies beyond the perturbation of the segrosome: (1) metabolic and/or physiological alterations, and (2) the perturbation of pathogenicity systems/factors indirectly triggered by the disruption of *parB*. To eliminate such possibilities, we began by examining the mutants under the microscope in order to detect any morphological changes in the cells that could support a segregation defect.

**Figure 4:**
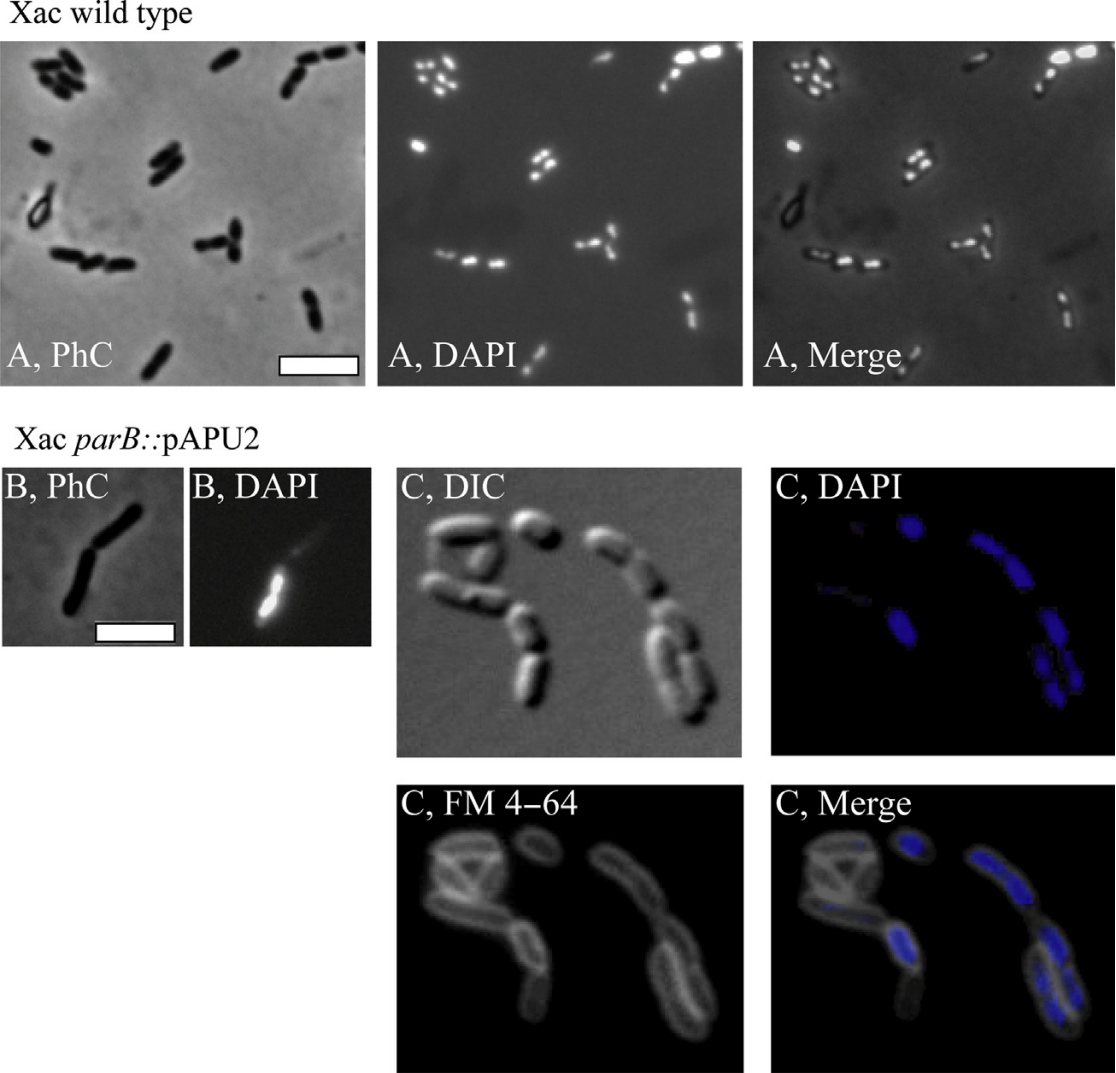
Morphological analysis of Xac *parB*::pPAU2 mutant cells. The Xac *parB*::pPAU2 mutant, which expresses only truncated forms of ParB, was compared with Xac wild type using various combinations of phase contrast (PhC), differential interference contrast (DIC), and fluorescence microscopy; the membrane and nucleoid stains FM 4–64 and DAPI, respectively, were used as indicated. Wild-type and mutant cells were cultivated in NYG medium at 30°C, and inspected around the OD_600 nm_ ˜0.3; cells were immobilized onto 1% agarose-covered slides. (A) Xac wild type. (B–C) Xac *parB*::pPAU2. Scale bar corresponds to 4 μm.

**Figure 5:**
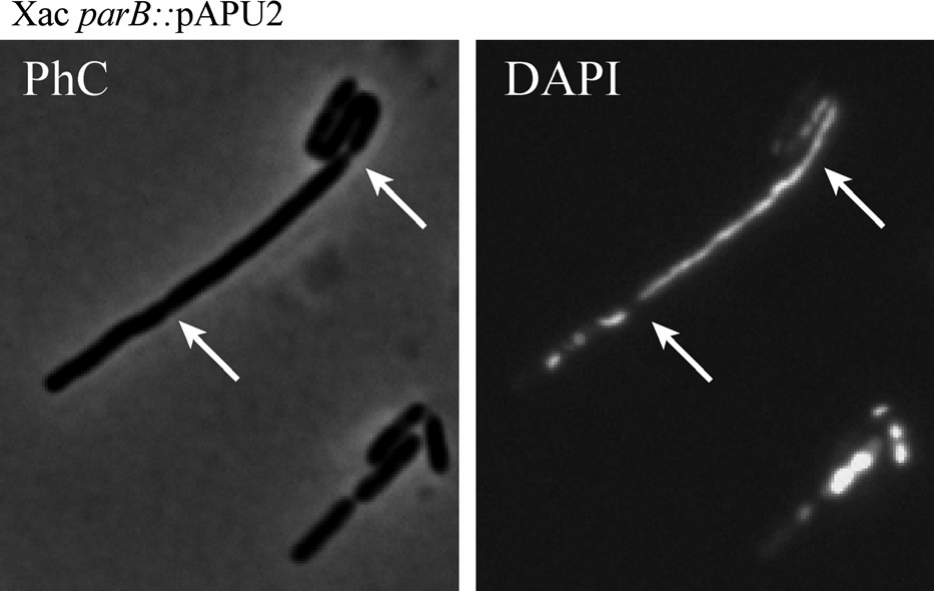
The filamentation phenotype of Xac *parB* mutant. The Xac *parB* mutant (Xac *parB*::pPAU2) was investigated under phase contrast (PhC) and fluorescence microscopy of DAPI-stained cells (DAPI) in order to evaluate their chromosome organization. Here, we show the picture of a representative filament from cells cultivated in NYG medium at 30°C, and visualized around the OD_600 nm_ ˜0.3; arrows show the closure of septa; on the right (DAPI), septum is closing without chromosome clearance. Scale bar corresponds to 4 μm.

In general, cultures of the *parB* mutant Xac *parB*::pAPU2 had a mix of filaments and rods (Figs. 4, 5). Percentages of the entire cell types documented are shown in Table 2. Filaments and/or irregular chains were approximately 5% of the cell types in a culture (*n* = 400) (Fig. 5). Close inspection of these filamented cells showed the presence of septal constrictions (arrows in Fig. 5), in which the division pattern displayed seems as if a particular rod started to grow and divide normally, losing the ability to form septa in successive cellular cycles; thus, filamentation seems to happen with elder rods attached to their tips. Despite the propensity to grow in long filaments, the division machinery seems operative in these mutants.

**Table 2:** Morphological analysis of Xac *parB*::pAPU2.

	Cell length (*n* = 200), *μ*m	Filaments (*n* = 400)	Anucleate	Minicells
Xac wild type	1.44 ± 0.31	0	0	0
Xac *parB*::pAPU2	2.56 ± 0.42*	5%	30%	0

Data correspond to the average cell length ± standard deviation.

* *P* < 0.05; One-way ANOVA with Tukey posttest.

Considering the nonfilamented cells of Xac *parB*::pAPU2, they looked apparently longer than the wild-type strain (compare Fig. 4A and B; Table 2). In order to quantitatively evaluate this, we measured 200 individuals of each culture (wild-type and Xac *parB*::pAPU2), and calculated the average cell length. We observed significant differences between them: the average wild-type cell length was 1.44 ± 0.31 *μ*m, whereas the Xac *parB*::pAPU2 mutants measured 2.56 ± 0.42 *μ*m.

To subsequently estimate the effects of *parB* disruption on the chromosome organization of Xac *parB*::pAPU2, cells were labeled with 4′,6-diamidino-2-phenylindole (DAPI) and visualized by fluorescence microscopy (Figs. 4, 5). Cultures of Xac *parB*::pAPU2 exhibited an increase in the number of anucleate rods (˜30% of the cell types, Fig. 4B and C), which contrasts with an absence of DNA-free cells in cultures of wild-type Xac (Table 2). When we looked at filamented cells of the Xac *parB* mutant, we observed a considerable accumulation of chromosomal mass, sometimes adopting the form of a continuum in which the nucleoid fills up the whole of a cell compartment extending through and preventing the closure of a septum (Fig. 5; arrow on the right). Minicells were not detected neither in cultures of Xac wild-type nor in the *parB* mutant (Table2). Altogether, data show that the disruption of *parB* in Xac leads to a severe impairment of the chromosome segregation process, which also culminated in an unexpected alteration of its ability to stimulate citrus canker symptoms.

### Subcellular localization of ParB-GFP

**Figure 6:**
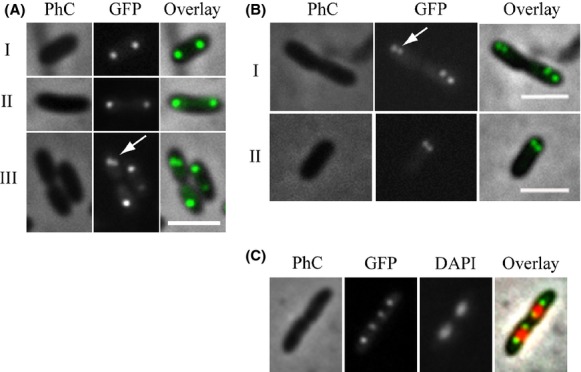
Localization of ParB-GFP. The localization pattern of ParB-GFP was analyzed in different cell types of a Xac *parB*::pAPU3 expressing ParB-GFP from the native *pparAB* promoter. (A, B, C) represent cells photographed during different moments of the cell cycle; arrows mark recently divided origins of replication (see text). Cells were cultivated in NYG medium at 30°C until the OD_600nm_ of ˜0.3, immobilized onto 1% agarose-covered slides, and visualized using fluorescence microscopy. Scale bar corresponds to 4 μm. PhC, phase contrast; DAPI, nucleoid stain.

The localization patterns of several chromosomally encoded ParB-like proteins have been documented in vivo and it constitutes an excellent indicative of function in chromosome segregation (Glaser et al. 1997; Mohl and Gober 1997; Jakimowicz et al. 2005; Fogel and Waldor 2006; Bartosik et al. 2009; Maloney et al. 2009; Donovan et al. 2010). To further extend our characterization of Xac ParB, we prepared a mutant strain, Xac *parB*::pAPU3, which expresses ParB-GFP under the control of *pparAB* at physiological levels (Fig. 6) (Xac *parB*::pAPU3 was constructed using the same strategy depicted above for the expression of ParB-TAP). In Figure 6A (I and II), we show the localization of ParB-GFP in cells without a clear sign of septal constriction. Note the presence of two foci per compartment, each occupying one of the cellular poles. This localization pattern was very similar to the one documented for the ParB homologue of *C. crescentus* (Mohl and Gober 1997). Inspection of several fields revealed another localization pattern in which the ParB-GFP focus is splitting in two in just one of the cellular compartments (Fig. 6A-III, arrow), whereas in the other it apparently remains as a single entity. The pattern seen in Figure 6A-III is probably a resultant of either the initiation of a new replication event in only one of the cell compartments, at a late stage of septation (which implies an asynchrony in the process of initiation of chromosome replication), or the brighter individual focus would in fact be two ParB-GFP foci superimposed. We favor the latter possibility as we also observed localization patterns in which the cells seemed to be in synchrony with regard to the initiation of replication (Fig. 6B). In Figure 6B-I, we see dividing cells and two closely positioned ParB-GFP foci occupying in each compartment just one of the polar regions. Here, new replication events that would separate the origin regions of the chromosomes are taking place at the poles; hence, the replisomes should be nearby. The cellular type shown in Figure 6B-II (a nondividing rod) seems to support the idea that the origins in Xac initiate replication toward one of the cellular poles, which is similar to the replication patterns exhibited by *C. crescentus*, *V. cholerae*, and *C. glutamicum* (Mohl and Gober 1997; Fogel and Waldor 2006; Donovan et al. 2010). Finally, we performed DAPI staining of cells and captured individuals in late stages of septum closure judged by the placement of the ParB-GFP foci within the cells (Fig. 6C). We observed four ParB-GFP foci evenly distributed inside the two linked cell compartments, where ParB-GFP colocalizes with the edges of the nucleoids. If we consider that these edges comprise the origins of replication, similar to *B. subtilis* and many other systems (Leonard et al. 2005b; Mierzejewska and Jagura-Burdzy 2012), *parS* in Xac is located in this region (at ˜3 kb from the origin in the chromosome of Xac (Livny et al. 2007)) and ParB-GFP is expected to be bound to it.

**Figure 7:**
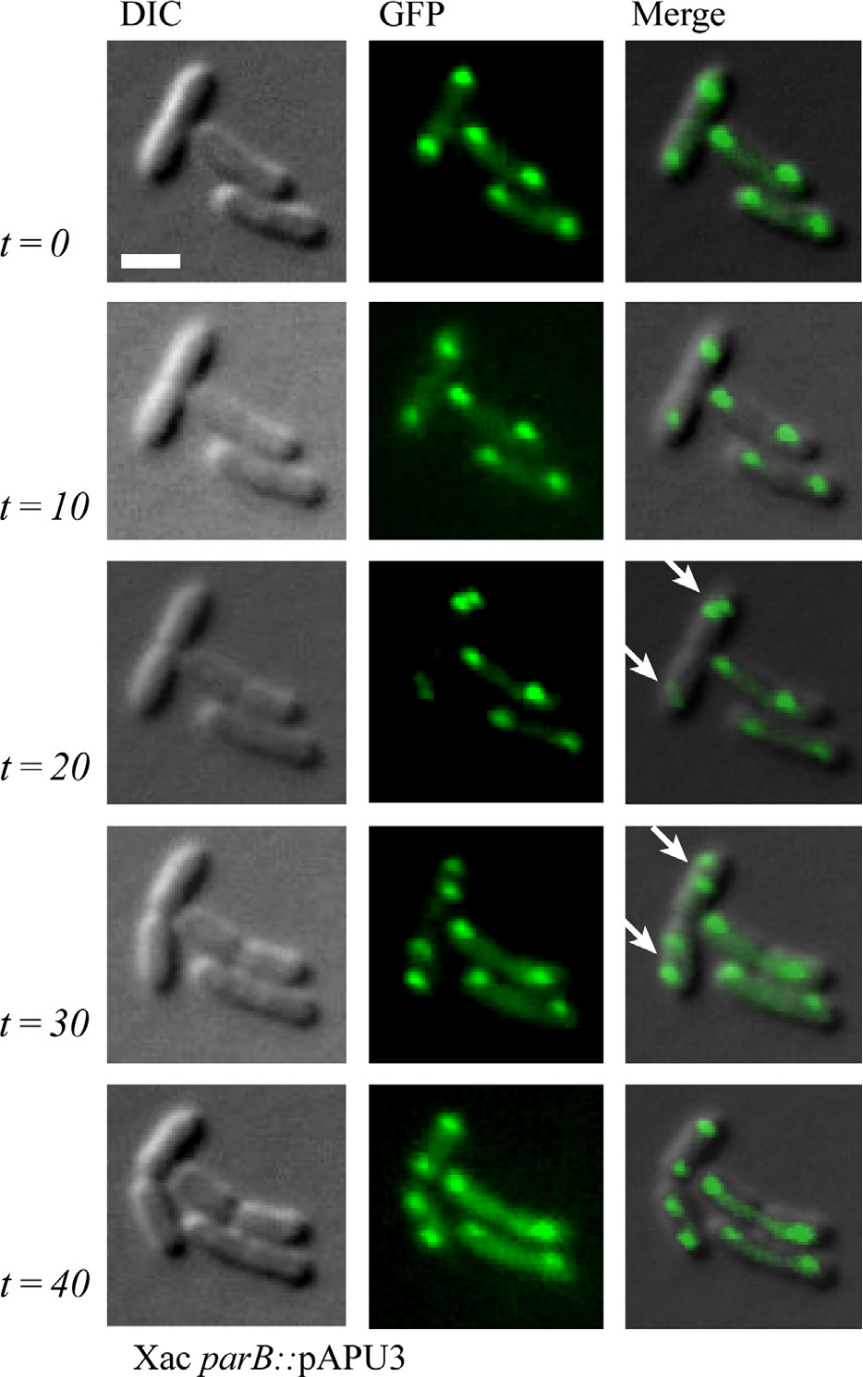
Time-lapse microscopy of Xac *parB*::pAPU3. The localization of ParB-GFP in Xac *parB*::pAPU3 evidences an asymmetric mode of chromosome partitioning. Cells were cultivated in NYG medium at 30°C until the OD_600nm_ of ˜0.3, and photographed at 0, 10, 20, 30, and 40 min as indicated. Arrows mark cells in which the replication origins have divided near the cellular poles (see text). DIC, differential interference contrast microscopy. Scale bar corresponds to 4 μm.

In Figure 6B-II, we showed evidence that Xac initiates chromosome replication close to a cellular pole. This suggests that chromosome segregation is asymmetric in this plant pathogen as it was also proposed for *C. crescentus*, *V. cholerae*, and *C. glutamicum* (Fogel and Waldor 2006; Donovan et al. 2010; Ptacin et al. 2010). In order to further characterize the asymmetric pattern of chromosome segregation in Xac, we performed time-lapse microscopy using Xac *parB*::pAPU3 (Fig. 7). From *t* = 0 to *t* = 20 min the leftmost dividing rods started new chromosomal replication events that can be seen by the division of the ParB-GFP foci (arrows in *t* = 20 min). Note that at the beginning of the process (from *t* = 0 until *t* = 20), the origins of replication, and consequently the ParB-GFP foci associated with them, were situated near the cellular poles and opposite to the septum. From *t* = 20 to *t* = 40, we see the innermost origins/ParB-GFP foci moving toward the septum until they reach the region that will become the new pole after cytokinesis. Therefore, we conclude that Xac exhibits asymmetric chromosome segregation.

## Discussion

We demonstrated recently that esters of gallic acid perturbed the cell division and/or the chromosome segregation apparatuses of Xac. Thus, these compounds constitute promising antimicrobial agents against citrus canker and probably other bacterial diseases (Silva et al. 2013). Here, we further characterized the Xac mutant strain used by Silva et al. (2013) that is labeled for the centromere (Xac *parB*::pAPU3), and extended the studies with ParB encoded by this plant pathogen. First, we showed that Xac ParB is stably expressed and operates on chromosome segregation. Chromosomally encoded ParB-like proteins have been characterized in several bacteria (recently reviewed by (Mierzejewska and Jagura-Burdzy 2012)), where the perturbation of their normal function and/or their cellular dosage with respect to their associated ParA partners often lead to an increase in the number of anucleate cells, cell filamentation, and nucleoid disorganization. Disruption of *parB* in Xac produced these classical phenotypes (Figs. 4, 5). In addition, in the absence of full-length ParB, Xac was not able to colonize the host citrus (Fig. 3). Second, the subcellular localization of ParB-GFP suggests an asymmetric mode of replicon segregation in Xac, which resembles the chromosome segregation patterns observed for *C. crescentus*, *V. cholerae*, and *C. glutamicum* (Fogel and Waldor 2006; Donovan et al. 2010; Ptacin et al. 2010). This, along with the fact that lack of ParB produced a remarkable cell filamentation phenotype suggests that ParB may be involved with cell division in Xac.

The inability to produce disease symptoms *in planta* documented for the Xac *parB* mutant is a novel effect, which reinforced our previous observations that alkyl gallates that were able to perturb the subcellular localization of ParB-GFP also precluded the ability of Xac to colonize citrus (Silva et al. 2013). In the past decade, several studies gradually unveiled the participation of ParAB proteins with various cellular processes other than chromosome segregation. In *P. aeruginosa*, for example, absence of ParB altered bacterial growth and affected swarming and swimming motilities (Lasocki et al. 2007; Bartosik et al. 2009). Slower growth rates were also detected in our experiments for the *parB* mutant of Xac. In *C. crescentus*, it was demonstrated that ParB operates on cell division by sequestering the FtsZ inhibitor MipZ to areas away from the cell center where the divisome should assemble (Thanbichler and Shapiro 2006b; Kiekebusch et al. 2012). In the Gram-positive bacterium *B. subtilis* the ParB-like protein Spo0J recruits structural maintenance of chromosomes to the origin of replication where it was proposed to assist in the organization of the region to enable more efficient chromosome segregation (Sullivan et al. 2009). Although Spo0J may help in replicon partition, it is required for sporulation in *B. subtilis*, and recently it has also been implicated with the control of initiation of DNA replication by modulating the action of the ParA-like factor Soj (Ireton et al. 1994; Scholefield et al. 2011). The involvement of ParAB proteins with control of DNA replication was also reported for *V. cholerae* (Kadoya et al. 2011). We are not certain yet whether the pathogenicity shutdown following *parB* disruption in Xac has any association with pathogenicity systems/routes encoded by the bacterium. We have employed yeast two-hybrid analyses in an attempt to identify interactions of ParB with factors belonging to known pathogenicity systems in Xac (A. Ucci, S. C. Farah, and H. Ferreira, unpubl. results); but so far, nothing was detected. Therefore, we believe that the loss of virulence may well be simply a response to the perturbation of the vital cellular processes of chromosome segregation and/or cell division.

In this work, we showed evidence for asymmetric chromosome segregation in Xac (Figs. 6, 7). First, we see newly duplicated origins of replication labeled with ParB-GFP occupying just one of the cellular poles (Figs. 6B-I, 7). Later, the two ParB-GFP foci are well separated from each other within the same cellular compartment (Figs. 6C, 7), which suggests that segregation occurs with the internally located origins migrating from the point of duplication toward the opposite pole (Fig. 7). Asymmetric chromosome segregation was documented for *C. crescentus*, *V. cholerae*, and *C. glutamicum* as well (Fogel and Waldor 2006; Donovan et al. 2010; Ptacin et al. 2010). For the most extensively characterized system, *C. crescentus*, the chromosome is oriented such that the origin of replication (designated *cori*) is close to what is called the old pole (*C. crescentus* is known for exhibiting a strict polar asymmetry, in which diverse protein factors/systems are arranged in one but not at both cellular poles at once). Before DNA replication starts, ParB is bound to *parS* located around *cori*; ParB/*parS* organizes a centromere (Toro et al. 2008) that is kept anchored to the old pole by PopZ (Bowman et al. 2008; Ebersbach et al. 2008). When replication fires, the centromere is duplicated, and one copy of this region is captured by ParA filaments that come from the opposite cell pole, where they are anchored to TipN (a factor that marks the new pole) (Ptacin et al. 2010; Schofield et al. 2010). ParB/*parS* then interacts with ParA, ParB stimulates ParA depolymerization that shortens the filament, which is followed by subsequent recapture of ParB/*parS*; orientation of the centromeric region to the new pole operates chromosome partitioning. A related segregation mechanism was also proposed for *V. cholerae* (Fogel and Waldor 2006), which presents some differences with that of *C. crescentus*, one of which is the use of a distinct polar anchor designated HubP, which interacts with ParA (Yamaichi et al. 2012). As for *C. glutamicum*, DivIVA was demonstrated to be the factor that tethers the ParB/DNA complex at the cell pole (Donovan et al. 2012). Considering the similarities between systems, in particular the ParB-GFP subcellular localization patterns documented for *C. crescentus*, *V. cholerae*, *C. glutamicum*, and Xac, we expect to identify protein factors of equivalent functions in Xac in the near future.

The localization pattern of ParB-GFP in Xac as well as the observation that chromosome segregation and cell division may be interlinked raised the idea that Xac could control septum placement in a manner similar to *C. crescentus* (Thanbichler and Shapiro 2006b). Curiously, similar findings and suggestions were recently reported for *M. smegmatis* (Ginda et al. 2013), where deletion of *parA* produced aberrant phenotypes of chromosome segregation and septum positioning leading to cell elongation. The association of chromosome segregation and cell division in Xac was first proposed by Silva et al. (2013), who demonstrated that alkyl gallates able to perturb the localization of ParB-GFP in this bacterium apparently acted on septum assembly as well. In *C. crescentus*, septum site selection depends on the centromere migration dynamics (Thanbichler and Shapiro 2006a), where MipZ, an inhibitor of FtsZ polymerization, interacts and localizes with ParB bound to *parS* (Thanbichler and Shapiro 2006a; Kiekebusch et al. 2012). As the centromeric complexes occupy the poles, the FtsZ inhibitor MipZ is kept away from the cell center where the septum is assembled. Therefore, disruption of ParB in *C. crescentus* perturbs the control of septation and leads to cell filamentation. Xac does not have an obvious *mipZ* homologue on its genome. MipZ is conserved in *α*-proteobacteria, and belongs to the Mrp/MinD family of P-loop ATPases, being structurally related to the ParA superfamily members Soj and MinD (Kiekebusch et al. 2012). However, Xac carries at least four *parA*-like ORFs (XAC0192, XAC1907, XAC2205, and XAC2433) that could have a *mipZ* function, which we shall investigate experimentally soon. Another possibility would be that Xac utilizes other protein factors for septum site selection. Noteworthy, the genome of this plant pathogen has MinCDE coding regions. The MinCD system, which operates division site selection, has been well studied in *B. subtilis* and *E. coli*, both bacteria displaying symmetrical chromosome segregation (reviewed by Lutkenhaus 2007). We are in the process of characterizing if and how Xac coordinates chromosome segregation with cell division utilizing MinCD. Ultimately, the filamentation observed for the Xac *parB* mutant strain could be caused by the action of a nucleoid occlusion system, analogous to the Noc/SlmA systems of *B. subtilis* and *E. coli* (Wu and Errington 2004; Bernhardt and de Boer 2005). As illustrated in Fig. 5, chromosome replication in the Xac *parB* mutant, followed by inadequate segregation, apparently produces cells with several chromosomal copies that if associated with factors able to inhibit septum assembly/closure would lead to filamentation. However, Xac does not seem to encode any Noc/SlmA-like protein that could fulfill the function of a nucleoid occlusion factor (Wu and Errington 2004; Bernhardt and de Boer 2005).

## Supporting Information

Figure S1 Growth curve of the mutant Xac *parB*::pAPU2. Bacteria were cultivated for 36 h in NYG medium at 30°C and 200 rpm. Each point in the graph corresponds to an average of optical densities (OD_600nm_) calculated from three independent experiments; vertical bars indicate standard deviation values calculated for each average. Here, we show the measurements done for mutant 3; however, mutants 1 and 2 exhibited similar growth patterns. Blue, wild-type Xac; red, Xac *parB*::pAPU2.

Table S1 Oligonucleotides.
