# Supplementary material for: Asymmetric chromosome segregation in Xanthomonas citri ssp. citri
Source: Microbiologyopen. 2013 Dec 15;3(1):29–41. doi: 10.1002/mbo3.145 (PMC3937727; doi:10.1002/mbo3.145)
Supplement: Figure S1 — Growth curve of the mutant Xac parB::pAPU2. Bacteria were cultivated for 36 h in NYG medium at 30°C and 200 rpm. Each point in the graph corresponds to an average of optical densities (OD600nm) calculated from three independent experiments; vertical bars indicate standard deviation values calculated for each average. Here, we show the measurements done for mutant 3; however, mutants 1 and 2 exhibited similar growth patterns. Blue, wild-type Xac; red, Xac parB::pAPU2. [file mbo30003-0029-sd1.pdf]

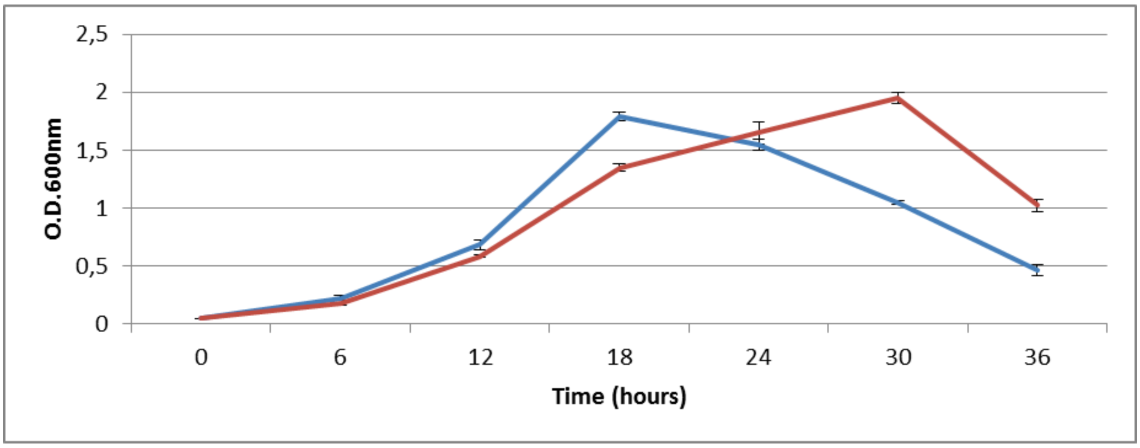


**Figure S1** Growth curve of the mutant Xac *parB*::pAPU2. Bacteria were cultivated for 36h in NYG-medium at 30 $^{o}$C and 200 rpm. Each point in the graph corresponds to an average of optical densities (O.D.$_{600\ nm}$) calculated from three independent experiments; vertical bars indicate standard deviation values calculated for each average. Here we show the measurements done for mutant 3, however, mutants 1 and 2 exhibited similar growth patterns. Blue, wild-type Xac, red, Xac *parB*::pAPU2.
