# Supplementary material for: Asymmetric chromosome segregation in Xanthomonas citri ssp. citri
Source: Microbiologyopen. 2013 Dec 15;3(1):29–41. doi: 10.1002/mbo3.145 (PMC3937727; doi:10.1002/mbo3.145)
Supplement: Table S1 — Oligonucleotides. [file mbo30003-0029-sd2.pdf]

Table S1. Oligonucleotides.

Name	Sequence*
PBS1479F	5´-TGAGGATCCATGGAAAAGAGAAGATGGAAAAAG
PBS1479R	5´-ATTCTAGATCAAGCTTCAGGTTGACTTCCCCGCGG
parBintF	5´-GATAGCTTGCGCCAGTTGC
parBintR	5´-CCTGTGGGGCCACCGGCGC
ParBF20070822	5´- ATGGATCCATGAACAAGCCGATCCCCGCAAAGAAG
ParBR20080530	5´- ATGGATCCGCTACGCTGCATGCGCAGGC
ParB20100309	5´- ATCTCGAGACCCAAGCTACGCTGCATGCGCAGGC

* The restriction sites used for cloning are underlined
